# Supplementary material for: Spatial signals link exit from mitosis to spindle position
Source: eLife. 2016 May 11;5:e14036. doi: 10.7554/eLife.14036 (PMC4887205; doi:10.7554/eLife.14036)
Supplement: Supplementary file 1. — The strains listed in Supplementary file 1 are all derivatives of W303. The table shows the relevant genotype for each yeast strain and the associated strain number. DOI: http://dx.doi.org/10.7554/eLife.14036.019 [file elife-14036-supp1.docx]

Supplemental File 1: Strains Used in this Study

| A1411 | *MATa, CDC14-3HA.* |
| --- | --- |
| A2587 | *MATa ade2-1 leu2-3 ura3 trp1-1 his3-11,15 can1-100 GAL phi+ (wild-type W303)* |
| A32922 | *MATa, ura3::pAFS125-TUB1p-GFPTUB1::URA3, dyn1::URA3* |
| A33138 | *MATa, ura3::pAFS125-TUB1p-GFPTUB1::URA3* |
| A33729 | *MATa, kar9::HIS5, ura3::pAFS125-TUB1p-GFPTUB1::URA3* |
| A34722 | *MATa, pRS306-pCTS1-2xmCherry-SV40NLS::URA3, ura3::pAFS125-TUB1p-GFPTUB1::URA3, dyn1::HIS5Mx6* |
| A34832 | *MATa, pRS306-pCTS1-2xmCherry-SV40NLS::URA3, ura3::pAFS125-TUB1p-GFPTUB1::URA3, Dyn1-3V5-AID:KanMX, leu2::pTEF1-osTIR::LEU2, kar9::HIS5* |
| A35143 | *MATa, Dyn1-3V5-AID:KanMX, leu2::pTEF1-osTIR::LEU2, kar9::HIS5, pRS306-pCTS1-2xmCherry-SV40NLS::URA3, ura3::pAFS125-TUB1p-GFPTUB1::URA3, cdc3::mCherry-CDC3::URA3* |
| A35570 | *MATalpha, Dyn1-3V5-AID:KanMX, leu2::pTEF1-osTIR::LEU2, kar9::HIS5, cdc3::mCherry-CDC3::URA3, prm3::kanMX6* |
| A35571 | *MATa, Dyn1-3V5-AID:KanMX, leu2::pTEF1-osTIR::LEU2, kar9::HIS5, ura3::pAFS125-TUB1p-GFPTUB1::URA3, prm3::kanMX6* |
| A35699 | *MATa, leu2::pTEF1-osTIR::LEU2, ura3::pAFS125-TUB1p-GFPTUB1::URA3* |
| A35700 | *MATa, Dyn1-3V5-AID:KanMX, leu2::pTEF1-osTIR::LEU2, kar9::HIS5, ura3::pAFS125-TUB1p-GFPTUB1::URA3, spo12::HIS3* |
| A35707 | *MATa, Dyn1-3V5-AID:KanMX, leu2::pTEF1-osTIR::LEU2, kar9::HIS5, ura3::pAFS125-TUB1p-GFPTUB1::URA3* |
| A36028 | *MATa, Dyn1-3V5-AID:KanMX, leu2::pTEF1-osTIR::LEU2, kar9::HIS5, ura3::pAFS125-TUB1p-GFPTUB1::URA3, slk19::kanMX6* |
| A36264 | *MATa, Dyn1-3V5-AID:KanMX, leu2::pTEF1-osTIR::LEU2, kar9::HIS5, ura3::pAFS125-TUB1p-GFPTUB1::URA3, cdc15::CDC15-as1(L99G)::URA3* |
| A37463 | *MATa/alpha, Dyn1-3V5-AID:KanMX/ Dyn1-3V5-AID:KanMX, leu2::pTEF1-osTIR::LEU2/ leu2::pTEF1-osTIR::LEU2, kar9::HIS5/ kar9::HIS5, ura3::pAFS125-TUB1p-GFPTUB1::URA3, cdc14::cdc14-tdTomato::HIS3* |
| A37464 | *MATa/alpha, Dyn1-3V5-AID:KanMX/ Dyn1-3V5-AID:KanMX, leu2::pTEF1-osTIR::LEU2/ leu2::pTEF1-osTIR::LEU2, kar9::HIS5/ kar9::HIS5, ura3::pAFS125-TUB1p-GFPTUB1::URA3, cdc14::cdc14-tdTomato::HIS3* |
| A37610 | *MATa/alpha, Dyn1-3V5-AID:KanMX/Dyn1-3V5-AID:KanMX, leu2::pTEF1-osTIR::LEU2/ leu2::pTEF1-osTIR::LEU2, kar9::HIS5/ kar9::HIS5, ura3::pAFS125-TUB1p-GFPTUB1::URA3/ura3::pAFS125-TUB1p-GFPTUB1::URA3, spo12::HIS3/spo12::HIS3, cdc14::cdc14-tdTomato::HIS3* |
| A37753 | *MATa/alpha, Dyn1-3V5-AID: KanMX/ Dyn1-3V5-AID: KanMX, leu2::pTEF1-osTIR::LEU2/ leu2::pTEF1-osTIR::LEU2, kar9::HIS5/ kar9::HIS5, ura3::pAFS125-TUB1p-GFPTUB1::URA3/ura3::pAFS125-TUB1p-GFPTUB1::URA3, cdc14::cdc14-tdTomato::HIS3* |
| A37892 | *MATa, leu2::pTEF1-osTIR::LEU2, ura3::pAFS125-TUB1p-GFPTUB1::URA3, prm3::kanMX6* |
| A37895 | *MATa, Dyn1-3V5-AID:KanMX, leu2::pTEF1-osTIR::LEU2, kar9::HIS5, ura3::pAFS125-TUB1p-GFPTUB1::URA3, cdc14-1-AID::KANMX* |
| A38232 | *MATa/alpha, Dyn1-3V5-AID:KanMX/Dyn1-3V5-AID:KanMX, leu2::pTEF1-osTIR::LEU2/leu2::pTEF1-osTIR::LEU2, kar9::HIS5/kar9::HIS5, ura3::pAFS125-TUB1p-GFPTUB1::URA3* |
| A38233 | *MATa/alpha, Dyn1-3V5-AID:KanMX/Dyn1-3V5-AID:KanMX, leu2::pTEF1-osTIR::LEU2/leu2::pTEF1-osTIR::LEU2, kar9::HIS5,/kar9::HIS5, ura3::pAFS125-TUB1p-GFPTUB1::URA3, cdc14::cdc14-tdTomato::HIS3, cfi1::CFI1-GFP::KanMX6* |
| A38243 | *MATa, ura3::pAFS125-TUB1p-GFPTUB1::URA3, cdc14::cdc14-tdTomato::HIS3* |
| LY1043 | *MATa/alpha, swe1::kanMX/swe1::kanMX, PTUB1-GFP-TUB1-LEU2/TUB1, ZIP1-GFP(700)/ZIP1, Gal4-ER:URA3/Gal4-ER:URA3* |
